# Nanoplastics and Microplastics May Be Damaging Our Livers

**DOI:** 10.3390/toxics10100586

**Published:** 2022-10-04

**Authors:** Jianli Yin, Ye Ju, Honghao Qian, Jia Wang, Xiaohan Miao, Ying Zhu, Liting Zhou, Lin Ye

**Affiliations:** 1 Department of Occupational and Environmental Health, School of Public Health, Jilin University, Changchun 130021, China; 2 School of Public Health, Jilin University, Changchun 130021, China

**Keywords:** microplastics, nanoplastics, polystyrene microplastics, oxidative stress, liver injury

## Abstract

Plastics in the environment can be degraded and even broken into pieces under the action of natural factors, and the degraded products with a particle size of less than 5 mm are called microplastics (MPs). MPs exist in a variety of environmental media that come into contact with the human body. It can enter the body through environmental media and food chains. At present, there are many studies investigating the damage of MPs to marine organisms and mammals. The liver is the largest metabolizing organ and plays an important role in the metabolism of MPs in the body. However, there is no available systematic review on the toxic effects of MPs on the liver. This paper summarizes the adverse effects and mechanisms of MPs on the liver, by searching the literature and highlighting the studies that have been published to date, and provides a scenario for the liver toxicity caused by MPs.

## 1. Introduction

Global plastic annual production has increased from 1.7 million tons to 360 million tons over the past 70 years [1]. However, due to the production of large quantities of plastic products, low recycling rate, and poor management, plastics are widely present in the ocean, soil, air, and other environmental media with which human beings have close contact [2]. Under the action of physical erosion, biodegradation, or photocatalytic oxidation, plastics entering the environment can be degraded into plastic particles, and those particles with a particle size of less than 5 mm are called microplastics (MPs) [3]. Among them, MPs with a particle size of less than 1000 nm [4] or 100 nm [5] are called nanoplastics (NPs). In this paper, NPs refer to plastics with particle sizes not larger than 1000 nm. There are many kinds of MPs/NPs, such as polyethylene (PE), polypropylene (PP), polyvinyl chloride (PVC), polyamide (PA), polystyrene (PS), polyethylene terephthalate (PET), polymethyl methacrylate (PMMA), micro (nano)plastics [6], etc.

Studies have shown that MPs/NPs pollution exists in a variety of environmental media, including terrestrial oceans, rivers, lakes, and polar glaciers [7]. In the global marine environment, the MPs/NPs floating on the sea surface are mainly concentrated in five current circulation belts of the North Atlantic, South Atlantic, North Pacific, South Pacific, and Indian Ocean [7]. The abundance of MPs/NPs in seawater greatly varies in different regions and studies, ranging from 4.8 × 10^−6^/m^3^ to 8.6 × 10^3^/m^3^. In addition, the amount of plastic released into the soil each year is estimated to be 4 to 23 times that of the plastic released into the marine environment [8]. The abundance of MPs/NPs in the soil varies from a few to tens of thousands per kilogram (dry weight). Soils in other countries and regions in the world also face this phenomenon, such as Switzerland [9], Chile [10], Mexico [11], etc. MPs are also contained in the air. Dris et al. detected MPs for the first time in air deposition in Paris, France, with an abundance of 29~280/m^2^/d [12]. Both field investigations and laboratory studies have uncovered the phenomenon of microplastic transport at the trophic level, and these findings call for attention to the bioaccumulation, biomagnification, and toxic effects of microplastics on the organisms at a high trophic level [13,14].

It is generally believed that MPs/NPs enter the human body mainly through the respiratory tract [15,16] and the digestive tract [17,18]. They can also enter the body through the skin [19,20]. Airborne MPs/NPs can lead to respiratory exposures, while MPs/NPs in food, drinking water, and air deposition can lead to digestive system exposures. As early as 1998, Pauly et al. examined 114 human lung specimens and found fibers of up to 250 μm in length in 99 (87%) of these specimens [21]. This is the first report on the human inhalation of natural fibers and plastic fibers, and it also supports the fact that MPs can enter the human body through respiration. In 2018, Austrian scientists examined the stool of volunteers from 8 countries and found MPs in all the stool samples, confirming the exposure of the human digestive tract to MPs, with an average of 20 plastic particles per 10 grams of stool. There are as many as 9 types of MPs in stool samples, of which PP and PET are the most common [22]. A New York University study found that MPs are 20 times higher in infant feces than in that of adults, likely due to babies’ increased exposure to plastic due to their tendency to crawl on the floor, chew on plastic toys, and use plastic spoons and bottles’ middle. In addition, MPs with a size of 5~10 μm have also been detected in the human placenta [23]; in addition, in plastic-related occupational places, such as PVC production workshops, textile factory workshops, etc., people face higher concentrations of MPs exposure [16].

Many researchers have estimated human MPs intake due to single or multiple exposure routes by summarizing MPs contamination data in food and drinking water. For example, Van Cauwenberghe et al. estimated that Europeans could consume up to 11,000 MPs per person per year through shellfish intake [17]. Cox et al. compiled the data on MPs contamination from salt, seafood, honey, drinking water, and sugar, combined with the dietary habits of Americans, and estimated that the annual intake of microplastics per person was 39,000–52,000 [24]. For the MPs entering the human body from the upper respiratory tract, the human MPs/NPs exposure can be as high as 74,000–121,000 per year.

Currently, papers on MPs mainly focus on marine organisms. Studies have found that MPs can accumulate in the digestive tract [25,26] and other parts of marine organisms [27], as well as in rats [28] and mice [29]; for instance, the accumulation of MPs in the testes [30] and kidneys [29] of mice caused intestinal and liver dysfunction and was found to interfere with the immune response of the body and affect the function of the reproductive system. The liver is an organ with a metabolic function in the vertebrate body and plays the role of deoxidation, the storage of glycogen, and detoxification, which render it an important organ in human body. To date, there is no review on the effect of MPs on the liver. In this paper, by searching the published literature on MPs, we describe the damage of MPs to the liver and the relevant mechanisms.

## 2. Methods

The literature search followed the guidelines of PRISMA. First, we searched the keywords including both “microplastics” or “nanoplastics” and “liver” or “hepatic” on the PubMed or Web of Science until May 2022 (N = 231). Then, we excluded the duplicated references. Reviews and meta-analyses were also excluded (n1 = 17). Finally, we selected the studies for this review by browsing their abstracts (n2 = 214).

## 3. Keyword Co-Occurrence Analysis

**Figure 1:**
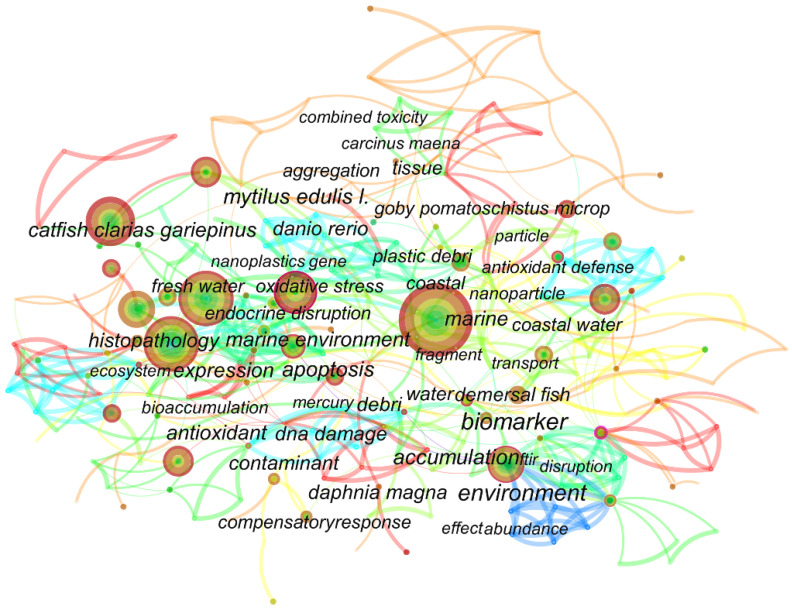
Keyword co-occurrence analysis.

Figure 1 shows the co-occurrence analysis of the keywords in eligible papers on the Web of Science. The results of the word frequency table (Table S1) showed that oxidative stress appeared 44 times, ranking third, and the first and second were microplastics and fish. Accumulation and bioaccumulation also occurred more frequently, with 32 and 16 occurrences, respectively. The high frequency of the appearance of such words as metabolism, inflammation, and lipid metabolism indicates that the current papers regarding MPs and NPs are focused on oxidative stress, inflammation, and metabolism.

## 4. Toxicity of MPs/NPs on the Liver

### 4.1. Internalization of MPs and NPs in Different Organisms

Internalization of MPs/NPs in soil plants: The discontinuous regions in plant roots, located at the root tips and secondary roots where the endoderm cells are immature, are known pathways of pathogen or bacterial infection; thus, MPs/NPs can directly internalize into the plant body through this cleft entry mode [31].

Internalization of MPs/NPs in phytoplankton and animals: Most of the MPs have a particle size close to the size of algal cells. Even if the particle size is much smaller than that of algal cells, they will hardly be internalized into algal cells due to the difficulty in penetrating the cell wall [32]. However, the latest research has found that some small-sized MPs do have the possibility to enter algal cells [33]. When MPs/NPs come into contact with algal cells, some of the MP/NP particles may be encapsulated by the plasma membrane’s microcapsules on the surface of the algal cells and become embedded in them [34]. Zooplankton can ingest MPs/NPs either actively (because of misjudging MPs/NPs as phytoplankton) or passively (because MPs/NPs are adsorbed on the surface of the phytoplankton or internalized into its cells) [35].

MPs/NPs that enter the organism through the digestive tract and respiratory tract enter the liver through intestinal absorption or epidermal infiltration and can also reach the liver through blood circulation [36]. MPs and larger NPs (greater than 200 nm) are not easily internalized, but 100 nm or smaller particles are easily endocytosed into cells [37]. Endocytosis is a key mechanism [38,39] by which cells take up NPs by wrapping them in vesicles or vacuoles that are pinch-off from their cytoplasmic membranes in an energy-dependent manner. These include clathrin-dependent endocytosis [40] and caveolin-dependent endocytosis [41]. Studies have shown that zebrafish hepatocytes were exposed to 5 mg/L and 50 mg/L of 65 nm PS-NPs, and PS-NPs were efficiently absorbed by ZFL and mainly accumulated in the lysosomes [42]. This indicates that 65 nm PS-NPs are internalized into the liver. In addition to endocytosis, internalization can also be performed in an energy-independent manner through passive diffusion [43].

### 4.2. Accumulation of MPs and NPs in the Liver

Current studies have shown that both NPs and MPs (25 nm [28]~90 μm [44]) can accumulate in the livers of marine fish [45] and mammals such as rats [28] and mice [46]. Moreover, whether MPs or NPs can accumulate in the liver and the amount of accumulation are closely related to their particle size. After the exposure of goldfish to 300 mg/L of PS-NPs and PS-MPs with particle sizes of 250 nm and 8 μm for 7 days, the accumulation rate of PS-NPs in the liver of goldfish was higher than that of PS-MPs [47]. The marine medaka was exposed to PS-MPs of 10 μm and 200 μm for 60 days, and PS-MPs of 200 μm were not detected in its liver [48]. Because larger MPs are easily filtered by the gills of marine organisms, smaller plastics, such as those of nanoscale, enter the bloodstream through the gills, initially accumulate in the gut, and then transfer to the liver [11]. Similar results were seen in PS-MPs exposed mice (1.46  ×  10^6^ items for 5 μm PS-MPs and 2.27  ×  10^4^ items for 20 μm PS-MPs via oral gavage), in which 5 μm PS-MPs accumulated in the kidney and gut more than 20 μm PS-MPs after 28 days of exposure [29]. Meanwhile, in vitro, PS-MPs of 1 μm can hardly enter HL7702 cells, while PS-NPs of 100 nm can enter hepatocytes and cause damage even at low concentrations [46]. Other studies [29,49] have also shown that PS-NPs are more likely to transfer and accumulate in tissues through circulation. Similarly, 65 nm PS-NPs can be absorbed by all zebrafish liver cells after 6 h of incubation, mainly accumulating in the lysosomes. Moreover, the internalization process presents a dose–response mode, that is, the higher the dose, the longer the incubation time, and the more PS-NPs in the cells [42]. However, this previous study did not examine the amount of PS-NPs taken up by zebrafish liver cells. Some studies [37,41] demonstrate that PS-MPs are not easily internalized by cells, while PS-NPs of 100 nm and below are easily taken up through the endocytic machinery. It was confirmed that the hepatic accumulation of MPs or NPs could produce toxic effects on hepatic function [50]. In addition, the toxic effect on hepatic function presented a size- and dose-dependent pattern [47]. The marine medaka was exposed to 10 mg/L PS-MPs of 10 μm and 200 μm for 60 days, and glucose metabolism and amino acid metabolism in the liver were affected; the levels of monosaccharides and amino acids in the 10 μm exposure group were significantly decreased, compared with those in the 200 μm exposure group. The reason for this difference in toxicity is closely related to the particle size of MPs. The particle size of 10 μm PS-MPs is much smaller than 200 μm. Compared with 200 μm PS-MPs, 10 μm PS-MPs are easier to enter the liver; thus, the impact of 10 μm PS-MPs on liver function is more serious than the effect of 200 μm PS-MPs [48]. The parameters and effects of the accumulation of MPs/NPs in the liver are shown in Table 1 and Table 2.

### 4.3. Liver Morphological Changes Caused by MPs and NPs

Both MPs and NPs can cause changes in the morphology of the liver to a certain extent, thereby affecting the normal function of the liver. Zebrafish were exposed to PS-MPs/NPs at 70 nm and 5 μm for 3 weeks, and hepatocyte necrosis, infiltration, and lipid droplets were observed in the 2000 μg/L group, suggesting that PS-MPs and PS-NPs can cause liver inflammation and hepatic lipid accumulation [27]. Liyun Yin et al. exposed the marine juvenile jacopever to PS-MPs of 15 μm (1 × 10^6^ particles/L) for 14 days, followed by a 7-day depuration period. In the exposed group, liver congestion was still seen in the fish, which was also confirmed with the analysis of pathological sections. The results also revealed that the damage to the fish liver by PS-MPs is continuous, and the liver damage is not only related to the particle size of MPs but also may be due to the negative charge of PS-MPs [59]. Besides PS-MPs, other kinds of MPs can also cause liver damage, such as PVC-MPs, PE-MPs, etc. [60,61,62]. After the European sea bass was exposed to 100 mg/kg and 500 mg/kg PVC-MPs or PE-MPs with a particle size of 40–150 μm for three weeks, compared with the control group, morphological changes in the hepatocyte and hepatocyte hypertrophy were observed in the exposed group. The void formation was significantly increased, and changes such as sinusoidal and vascular congestion presented among liver cells [62]. Similar results were seen in the tadpoles exposed to PE-MPs. Tadpoles were exposed to PE-MPs with a particle size of 35.46 ± 18.17 μm and a concentration of 60 mg/L for seven days, and in the exposed group, the livers of the tadpoles exhibited greater vasodilation, infiltration, hyperemia, hepatocyte edema-type degeneration, hypertrophy, and hyperplasia than those from the control group. In addition, it is worth noting that the tadpole hepatocyte nuclei exposed to PE-MPs have larger long and short axes, perimeters, areas, and volumes, all of which demonstrate the toxic effects of PE-MPs on the liver [61]. Clarias gariepinus was exposed to PVC-MPs (95.41 ± 4.23 μm) for 45 days with test diets containing 0.5%, 1.5%, and 3.0% PVC, followed by 30-day depuration. After exposure to PVC-MPs, the liver index increased, and glycogen depletion, fat vacuolization and degeneration, and hepatocyte necrosis occurred [62]. Increased liver body index was also observed in the groupers exposed to 20 mg/g of PS-MPs (22.3 μm) for 25 days, and the liver weight was significantly increased, suggesting that MPs could induce liver enlargement [50]. Goldfish were exposed to 10 μg/L, 100 μg/L, and 1000 μg/L of PS-NPs (70 nm) and PS-MPs (5 μm) for 7 days, and the ultrastructure showed increased hepatocyte interstitial space and mitochondrial vacuolation, which suggests that one of the targets of MPs may be mitochondria [63]. In addition to aquatic organisms, changes in liver morphology were also observed in the mice exposed to MPs. Male mice were exposed to 5 μm PS-MPs (20 mg/kg/day via drinking water) for 30 days. Compared with the control group, the mice from the PS-MPs group exhibited severe vacuolar degeneration and chronic inflammatory infiltration in the liver tissue, and hepatocyte edema [64]. In another study, mice were exposed to 100 μg/L and 1000 μg/L of PS-MPs (5 μm) for 6 weeks through drinking water. In the exposed group, the weight of the liver increased, and H&E staining displayed increased hepatic ballooning in the liver [65].

The above studies mainly highlight the effects of MPs on liver morphology, but NPs can also affect liver morphology in marine organisms and mammals. Zacco temminckii was exposed to 60 nm PS-NPs (5 mg/L) for 7 days. In the exposed group, the hepatocytes were destroyed and vacuolated, and the cell nuclei were aggregated and condensed [5]. Zebrafish were exposed to PS-MPs (10 mg/L) with a particle size of 100~120 nm, and after 7 days, hepatic necrosis and nuclear pyknosis were observed. After 35 days, eosinophilic granulomas, necrosis, and cytoplasmic degeneration appeared. Likewise, the fish exposed to 100 mg/L for 7 days exhibited liver histological changes, such as cytoplasmic vacuolation, nuclear pyknosis, and hepatocyte aggregation. On the 35th day, the liver sections displayed major inflammatory changes such as central venous congestion, cytoplasmic vacuolization, and hepatocyte degeneration. Different exposure doses and duration demonstrated that the degree of liver inflammation increased with dose and duration [66]. NPs can still cause pathological damage to the liver through the food chain. PS-NPs (1 mg/L) of 190 nm were transferred from Artemia franciscana to Larimichthys polyactis, and 8 days later, in the liver pathological section of Larimichthys polyactis, decreased liver tissue density and the band necrosis of hepatocytes were identified [67]. After mice were exposed to 100 nm PS-NPs for 60 days, histopathological examination revealed a concentration-dependent increase in PS-NPs-induced hepatocyte injury, including hepatocyte edema, enlarged nuclei, binucleated cells, irregular arrangement of hepatic cords, and portal inflammation [46]. The parameters and the liver morphological changes caused by MPs/NPs are shown in Table 3 and Table 4.

### 4.4. Changes in Liver Function Caused by MPs and NPs

The liver is the site of biotransformation and metabolism of many endogenous and exogenous compounds, and cytochrome P450 oxidase (CYP450) in the liver plays an important role in biotransformation and metabolism. In human hepatocytes, CYP450 is dominated by CYP1, CYP2, and CYP3. These three CYP450 compounds account for 70% of the total CYP450 in the liver and are involved in the metabolism of most drugs and toxicants. Through the action of enzymes, most endogenous compounds are biotransformed into more hydrophilic and polar compounds that can be excreted by the body. CYP450 isoenzymes are mainly involved in phase I reactions of oxidation, reduction, and hydrolysis in vivo, and can be induced and inhibited by exogenous compounds. Current research shows that CYP450 enzymes in the liver of marine organisms are affected by exposure to MPs and NPs. Zebrafish were exposed to 70 nm PS-NPs (0.5 ppm, 1.5 ppm, 5 ppm) for 7 days, and the expression of three CYP enzymes (CYP1A1, CYP11A1, and CYP19A1) was significantly increased in the liver of the zebrafish exposed to 1.5 ppm PS-NPs [51]. Jiannan Ding et al. exposed the red tilapia to 100 nm PS-NPs (1 μg/L, 10 μg/L, 100 μg/L) for 14 days, and the activity of CYP enzymes in the fish liver decreased first and then increased with time [71]. Enzymes such as ALT and AST are mainly present in the cytoplasm of hepatocytes but are released into the blood during liver injury. The activities of ALP, AST, and ALT in the plasma were increased after exposure to PS-NPs [52]. These results were consistent with the findings in the Wistar rat Rattus norvegicus [72].

Wei Cheng et al. differentiated embryonic stem cells into the liver organoids (LOs) and exposed to (0.25 μg/mL, 2.5 μg/mL, 25 μg/mL) 1 μm PS-MPs for 48 hours, and AST and ALT increased in the supernatant of LOs culture medium, meanwhile, the enzymatic activities of AST and ALT within the LOs were inhibited. It was demonstrated that PS-MPs could produce intracellular toxicity. When the LOs were exposed to varying doses of PS-MPs, the mRNA levels of the CYP450 family were upregulated. Among all the increased CYP450 family members, CYP2E1 was upregulated by the PS-MPs most remarkably [73]. Similar results for the upregulation of CYP450 enzymatic activity have been found in many studies [56,74]. Antònia Solomando et al. exposed Sparus aurata Linnaeus to 200–500 μm low-density polyethylene (LDPE-MPs) for 90 days, followed by depuration for 30 days. The activities of GSH-Px and GR and GST in the liver were significantly increased, with some recovery during the depuration [75]. This indicated that long-term sustained exposure is one of the important causes of liver toxicity.

## 5. Potential Mechanisms of MPs/NPs Toxicity on the Liver

### 5.1. Oxidative Stress

Growing evidence suggests that exposure to MPs/NPs is able to induce oxidative stress and produce oxidative damage in organisms [60,64,76,77] such as crabs, zebrafish, mice, etc. Oxidative damage is mainly manifested as changes in oxidative stress kinase activity, including SOD, CAT, GSH, GSH-Px, GR, GST, etc. The toxicity of MPs/NPs leads to the excessive production of reactive oxygen species (ROS) in the organism [78]. Excessive ROS can damage lipids in cells and lead to lipid peroxidation (LPO) [79]. Once the balance between the production and removal of ROS in the body deteriorates, the body will act through antioxidant enzymes such as SOD, CAT, and GPX to inhibit the development of LPO [80]. The main role of SOD is to catalyze the disproportionation of superoxide anion into oxygen and hydrogen peroxide, which is then catalyzed into H_2_O by CAT and GSH-Px enzymes [81]. GSH and GST facilitate the combination of glutathione and sulfhydryl transferase to form glutathione peroxidase, which is highly degradable to hydrogen peroxide [82]. In addition, GR exerts its detoxification effect by binding to glutathione-bound heterologous substances and catalyzing the reduction in GSSG to GSH [83]. Malonic dialdehyde (MDA) is one of the most important products of membrane lipid peroxidation, and the content of MDA is an important indicator reflecting the rate and intensity of lipid peroxidation [77].

**Figure 2:**
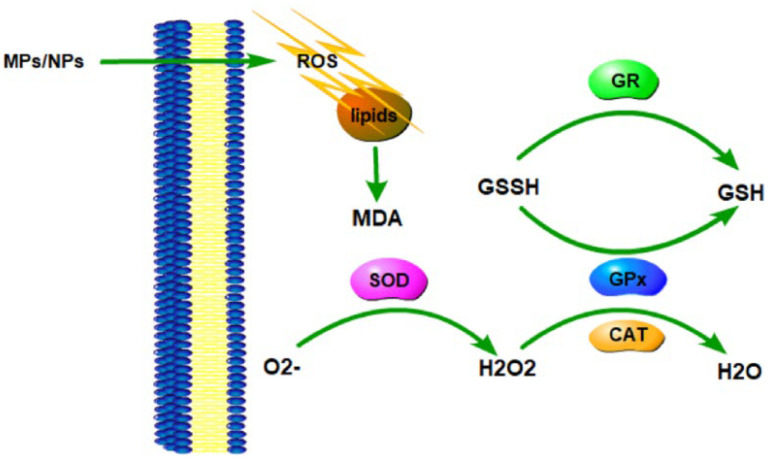
Schematic diagram of MPs/NPs-mediated oxidative stress response.

Compared with MPs, NPs have unique properties that aggregate more easily in living organisms than in the natural environment [84], and their aggregation is further influenced by the engineered function of nanoparticles or incidental coatings (such as fluorescent labels) and water chemistry [85,86]. This aggregation causes damage and induces a body response associated with increased reactive oxygen species, which is accompanied by a stronger oxidative stress response in the liver, further exacerbating the biotoxicity of NPs [87]. A recent study reported that treatment with 50 nm PS-MPs induced stronger oxidative stress and higher levels of antioxidant activation than that with 45 μm PS-MPs in the marine medaka [88] (Figure 2).

### 5.2. Inflammation

There are many studies showing a direct relationship between pollutants and inflammation [89]. Moreover, inflammatory responses also occurred in many organisms exposed to MPs/NPs, mainly manifested as increased expression of inflammatory factors and changes in the activities of enzymes related to inflammatory responses [90]. IL-1β and TNF-α are cytokines that promote inflammatory responses in the body [82], and IFN-γ is an antiviral cytokine that is mainly involved in mediating the immune and inflammatory responses [91]. When the body is exposed to pollutants, these cytokines are secreted from immune cells, mainly macrophages, to regulate the body’s inflammatory response [92,93]. MPO is a ferrous lysosomal enzyme involved in the removal of extracellular foreign matter [94]. The increase in the level of MPO in the body is usually associated with the infiltration of immune cells and the activation of inflammatory responses [95,96]. Nile tilapia were exposed to 350 nm and 9 μm PS-MPs for 28 days. The expression of the IFN-γ gene was upregulated in the fish exposed to 350 nm and 9 μm, and the expressions of IL8, IL-1β, and TNF-α genes were upregulated in the group of fish exposed to 9 μm, compared with the control [97].

**Figure 3:**
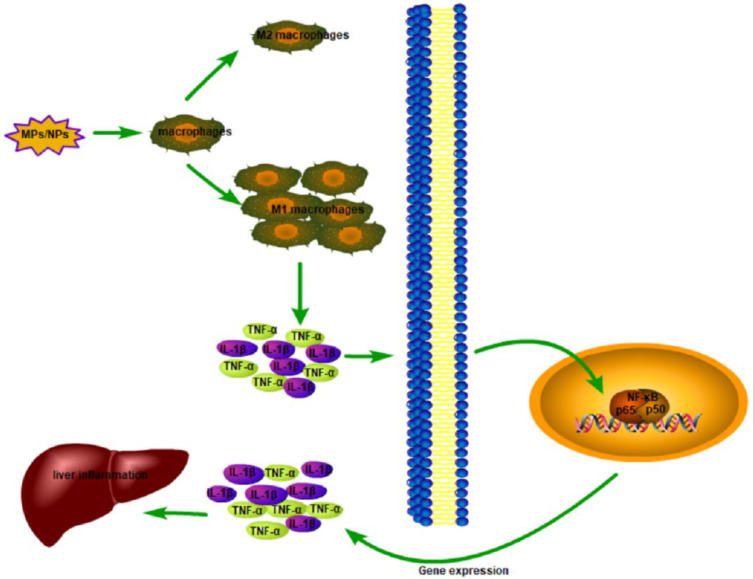
Schematic diagram of MPs/NPs-mediated inflammatory response.

Studies have shown that NPs can increase the infiltration of macrophages in the liver, upregulate M1 macrophages, and downregulate M2 macrophages. C57BL/6J mice were gavaged with 500 nm PS-NPs consecutively for 4 weeks (0.5 mg/day), and the percentage of macrophages and M1 macrophages were significantly increased after NPs exposure, while the percentage of M2 macrophages was significantly decreased [98]. Macrophages can be activated and differentiated into two different types of cells, M1 and M2, among which M1 mainly secretes proinflammatory factors and plays an important role in the early stage of inflammation, while M2 expresses the inhibiting inflammatory factors, which play a role in inhibiting the inflammatory response and repairing tissue in the body [99]. After injecting 500 nm PS-NPs in mice for 4 weeks, the expression of the inflammatory factors such as IFN-γ, TNF-α, and IL-1β in the liver was increased, and the levels of P65 and phosphorylated P65 proteins with the NF-κB pathway increased, indicating that NPs may activate the NF-κB signaling pathway in the liver [98] (Figure 3). The parameters and effects of liver inflammation caused by MPs and NPs are shown in Table 5.

### 5.3. Lipid Metabolism

MPs/NPs not only cause liver damage through oxidative stress and inflammation but also impair liver function by affecting liver lipid metabolism. Moreover, the effects of MPs on hepatic lipid metabolism were greater than those of NPs. The metabolic results of the zebrafish treated with PS-M/NPs showed that MPs and NPs could induce changes in 21 and 11 metabolites, respectively [27]. Compared with the NPs group, the crude fat in the fish liver of the MPs group was significantly reduced [106]. NPs may directly lead to liver injury and lipid accumulation, while MPs may trigger lipid metabolism disturbances by affecting gut microbial communities and homeostasis [102]. There are also gender differences in MPs/NPs on hepatic lipid metabolism. Liver lipid metabolism in female mice is more likely to be disrupted by MPs/NPs [65].

At present, there are few studies on the mechanism of abnormal liver metabolism after exposure to MPs/NPs, mainly focusing on the level of gene transcription and metabolomics. Studies have shown that MPs/NPs affect the expression of genes involved in lipid metabolism [105,107], such as peroxisome proliferator-activated receptor-alpha (PPARα) [107] and peroxisome proliferator-activated receptor-gamma (PPARγ) [52,102]. PPAR is involved in the regulation of fatty acid signaling as a key regulator of lipid metabolism, and it has three subtypes, namely PPARα, PPARβ/δ, and PPARγ [108]. PPARα regulates gene expression by binding to specific DNA sequences, leading to the transcriptional activation of target genes, such as apolipoprotein, lipoprotein lipase, and acyl-CoA oxidase, which are critical for lipid metabolism. In addition, PPARα has also been shown to regulate glucose metabolism, liver inflammation, and hepatocyte proliferation [109]. PPAR-γ is a ligand-activated nuclear transcription factor that plays a key role in fat absorption, storage, and metabolism [110]. After activation, PPAR-γ participates in lipid metabolism by regulating the expression of related genes. In addition, MPs/NPs can increase the mRNA expression of lipid-synthesis-related genes such as FAS, SREBP1, and PPARγ [102], as well as lipid transport genes such as CD36 and FATP1, and reduce the mRNA expression of lipid catabolism genes such as ATG1 and ACO [52]. Adenosine monophosphate-activated protein kinase (AMPK) plays an important role in regulating the homeostasis of lipid metabolism in the liver [111]. Studies have shown that MPs/NPs may cause lipid deposition in the liver through the inhibition of lipolysis mediated by the AMPK–PPARα signaling pathway [52].

**Figure 4:**
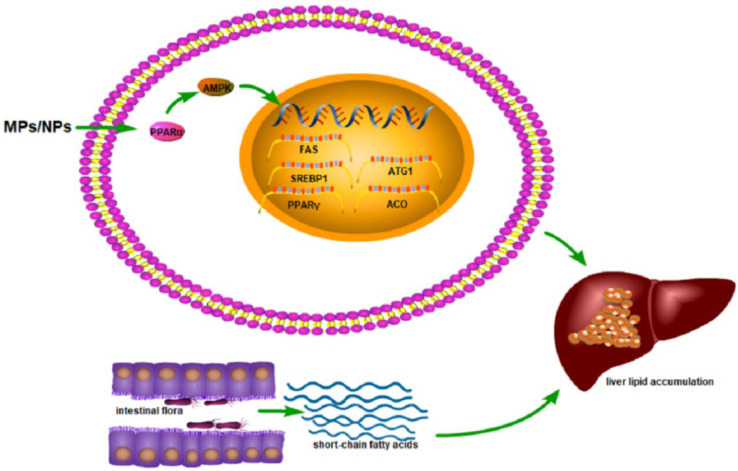
Schematic diagram of MPs/NPs-mediated hepatic lipid metabolism.

There are also studies showing that gut microbes may also affect lipid metabolism in the liver. Gut bacteria can produce short-chain fatty acids and then participate in lipid metabolism in the liver [112]. MPs/NPs affect the balance of gut microbes, which in turn affects lipid metabolism in the liver [102], which needs to be further explored. The metabolomic results showed that after exposure to MPs/NPs, liver metabolism significantly changed, mainly at the molecular level related to lipid metabolism, such as fatty acids, including monounsaturated fatty acids (MUFA), linoleic acid, FA-αH2, FA-ω-CH3, and fatty acyl chains, as well as choline, cholesterol, and amino acids, all of which are related to lipid metabolism [27]. Choline is an indispensable substance in the process of phospholipid synthesis and transport, which can promote lipid metabolism [113]. Leucine, isoleucine, and valine promote fatty acid metabolism [114], and exposure to MPs/NPs results in a reduction in these fatty acids [29] (Figure 4). The parameters and effects of the abnormal liver lipid metabolism caused by MPs and NPs are shown in Table 6.

### 5.4. Energy Metabolism

The liver is the center of energy metabolism and regulates energy storage through the biosynthesis or oxidation of fatty acids in animals [116,117]. Studies have shown changes in ATP/ADP/AMP metabolites in the liver after exposure to MPs/NPs in the zebrafish, indicating the disruption of energy metabolism in fish [27]. Similar results also suggest that ingestion of MPs depletes the energy reserves of marine worms and copepods [118,119] and affects the feeding activity of fish [120]. The nd5 gene is the core subunit encoding the NADH dehydrogenase (complex I) of the mitochondrial membrane respiratory chain, responsible for electron transfer in oxidative phosphorylation, a necessary process for ATP synthesis, and the mRNA levels of nd5 in fish changed after exposure to NPs, indicating a disturbance in the ability of fish to mobilize energy reserves [107]. It seems that MPs have a greater effect on energy metabolism in fish than NPs. The growth of fish after MPs/NPs exposure gradually decreased with the increase in particle size [106]. One study [106] showed that MPs treatment had a stronger inhibitory effect on the growth of fish than NPs treatment, and the energy reserve in fish after MPs exposure was less than that after of NPs exposure.

The levels of most monosaccharides and organic acids were significantly decreased in the liver of the medaka exposed to MPs, indicating that monosaccharide metabolism, tricarboxylic acid cycle, and glycolysis were inhibited in fish [48]. Moreover, the significantly lower levels of 6-phosphate gluconate and ribose in the fish liver indicated that the pentose phosphate pathway was inhibited, and nucleotide synthesis and NADPH production were affected, thereby affecting the energy supply in fish. After mice were exposed to MPs, the concentration of ATP related to energy metabolism in the liver decreased, and the LDH activity increased dramatically [29]. ATP levels and the LDH activity in the liver are related to the amount of energy in the liver [121]. An analysis of differences in serum metabolites between the exposed groups and the control group revealed that the changes in metabolites were related to compounds such as creatine, 2-ketoglutarate, and citric acid, which are vital for energy metabolism [122]. These results suggest that MPs exposure leads to energy deficit in mice.

After the body ingests MPs/NPs, MPs/NPs affect normal food intake and damage intestinal function, affect the absorption of nutrients in food, and lead to a decrease in energy in the body [123]. Moreover, the MPs/NPs entering the body affect the normal biological processes of the liver. The transcriptomic analysis of the livers of mice exposed to MPs revealed that multiple biological processes related to energy metabolism, such as glycolysis, glucose transport, fatty acid synthesis, and oxidation, were inhibited [65]. Similar results were found in MPs-exposed fish, MPs exposure also perturbed the metabolomic profile in the fish liver, with alterations in the metabolites mainly involving carbohydrates, fatty acids, amino acids, and nucleic acids. MPs exposure can also cause significant changes in most monosaccharide metabolic pathways, including galactose metabolism, fructose and mannose metabolism, pentose phosphate pathway, pentose and glucuronic acid interconversion, and glycolysis/gluconeogenesis [124].

The functions of the pentose phosphate pathway involve the production of sugar phosphates as biosynthetic intermediates and NADPH as a bioreductant [125], as well as several secondary function-dependent metabolites. Furthermore, glycolysis/gluconeogenesis is the main pathway related to energy metabolism. Thus, MP exposure triggers changes in energy metabolism [126]. The parameters and effects of the abnormal liver energy metabolism caused by MPs and NPs are shown in Table 7.

### 5.5. Programmed Cell Death

There are many ways of programmed cell death, including apoptosis, pyroptosis, ferroptosis, etc. Studies have demonstrated that these means of programmed cell death occur in the liver of MPs/NPs-exposed organisms.

#### 5.5.1. Apoptosis

**Figure 5:**
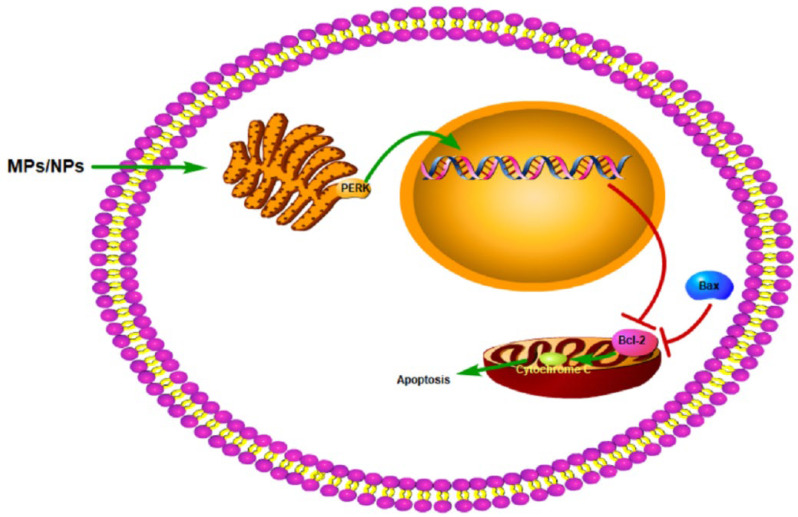
Schematic diagram of MPs/NPs-mediated hepatic apoptosis.

Studies have shown that when goldfish and grouper were exposed to PS-MPs, the level of hepatocyte apoptosis was significantly increased [50,128]. Similar results were found in mice. After mice were exposed to PS-MPs, the level of hepatocyte apoptosis was increased, mainly in the early stage [64]. Human SMMC-7721 cells also had elevated levels of apoptosis after exposure to NPs [129]. After exposure to PS-MPs, the Bax/Bcl-2 ratio and the level of caspase, a biomarker for detecting apoptosis in fish [130], were increased in the liver of zebrafish and sea bass [131]. In addition, the ratio of Bax/Bcl2 reflects the activation of procaspase and the occurrence of apoptosis [132]. After exposure to MPs, the expression of Bax and cytochrome C in human hepatocytes was significantly increased, and the expression of Bcl-2 was significantly decreased. After silencing the PERK gene in MPs-exposed human hepatocytes, MPs-induced mitochondrial apoptosis in L02 hepatocytes was attenuated, the expression of Bcl2 was increased, and the expression of Bax and cytochrome C was decreased, indicating that MPs may induce mitochondrial apoptosis through the PERK signaling pathway [133]. Nrf2 signaling is involved in the regulation of many endogenous signals in the body, such as autophagy and protein post-translational modification impairment [134]. As a phase II detoxification enzyme regulated by Nrf2, hepatocyte HO-1 is thought to play a key role in alleviating liver injury by inhibiting oxidative stress and apoptosis [135,136]. Studies have shown that the Nrf2/HO-1 pathway can exert a protective effect on the MPs-induced apoptosis of rat hepatocytes [64]. The effect of NPs on the apoptosis of hepatocytes was greater than that of MPs. Particles of smaller sizes induce higher levels of macrophage apoptosis in the zebrafish liver [137]. Similar results were seen in PE-MPs-exposed fish. The level of apoptosis in the fish liver was elevated after exposure to PE-MPs, and PE-MPs and small particle sizes were found to induce higher levels of apoptosis in the liver [138]. After NP exposure, the p38 MAPK signaling pathway was activated in RAW 264.7 cells and induced apoptosis [139]. In conclusion, MPs/NPs may induce hepatocyte apoptosis by activating PERK and MAPK (Figure 5). The parameters of the liver apoptosis caused by MPs and NPs are shown in Table 8.

#### 5.5.2. Pyroptosis and Ferroptosis

The NLRP3 inflammasome is the center of the intracellular regulation of inflammatory responses. NLRP3 is linked to caspase-1 via ASC and induces factor release and caspase-1-dependent pyroptosis [140]. This process causes proinflammatory cells to trigger the proteolytic cleavage of dormant procaspase-1 into active caspase-1, which converts the cytokine precursors pro-IL-1β and pro-IL-18, respectively, to mature and biologically active IL-1β and IL-18 [141]. Current studies have shown that the expression of ASC, caspase-1, and NLRP3 in the mouse liver induced by MPs exposure is significantly increased, and pyroptosis may be the key to MPs-induced damage to the liver tissue [104]. The light-chain subunit solute carrier family 7 member 11 (SLC7A11) plays an important role in ferroptosis, and GPX4 reduces potentially toxic lipid hydroperoxides (L-OOH) to nontoxic lipid alcohols (L-OH), thereby limiting the spread of lipid peroxidation within the membrane and preventing ferroptosis [142]. The expression of SLC7A11 and GPX4 decreased after MPs treatment, confirming that MPs may induce ferroptosis in the liver [104].

### 5.6. Other Mechanisms

In addition to the above-mentioned mechanisms, there are some mechanistic studies on endoplasmic reticulum stress and mitochondrial damage, and autophagy.

#### 5.6.1. Mitochondrial Damage

The exposure of goldfish to MPs/NPs induced vacuolation in the mitochondria of hepatocytes [63]. After exposure to MPs, the ultrastructure of the mouse liver showed mitochondrial cristae rupture [104] and mitochondrial vacuolization [64]. Mitochondrial DNA damage was also found in the livers of NPs-exposed mice [46]. Changes in mitochondrial morphology are regulated by dynamin-related protein 1 (Drp1) and mitochondrial fusion protein (Mfn2) [143]. After L02 cells were exposed to MPs, Drp1 expression was significantly upregulated, and Mfn2 expression was significantly downregulated [133]. The endoplasmic reticulum stress inhibitor 4PBA prevented the Drp1 upregulation and restored the protein expression of Mfn2 exposed to MPs. These results indicated that alleviating endoplasmic reticulum stress could effectively inhibit MP-induced mitochondrial fission.

Similar results were also seen in human liver cell lines. After human LO2 cells were exposed to 80 nm PS-NPs (0.0125, 0.125 mg/mL) for 48 h, the transmission electron microscopy analysis showed that NPs could enter cells and cause mitochondrial damage, resulting in excessive mitochondrial reactive oxygen species production [144]. Furthermore, the mitochondrial membrane potential was altered, and mitochondrial respiration was inhibited. These changes were observed at NP concentrations as low as 0.0125 mg/mL. Untargeted metabolomics confirmed that the most significantly affecting processes were mitochondrial-related. The metabolic functions of L02 cells were more susceptible to NP exposure than human lung epithelial BEAS-2B cells, especially at lower NP concentrations.

At present, there are few studies on the damage of MPs/NPs to the liver mitochondria, and there is some evidence that MPs/NPs damage the mitochondria of other organs. Human renal cortical proximal convoluted tubule epithelial cells (HK-2) can increase the levels of mitochondrial ROS and mitochondrial protein Bad after ingesting different concentrations of PS-MPs. MitoTEMPO is a mitochondrial ROS antioxidant that alleviates higher levels of mitochondrial ROS and Bad protein levels [145]. Intracellular mitochondria were damaged in rat basophilic leukemia (RBL-2H3) cells exposed to 50 nm PS-NPs [146].

#### 5.6.2. Autophagy

Microtubule-associated protein light chain 3 (LC3) is a major protein in the autophagy pathway and is the most widely used indicator of autophagosomes [147]. In addition, Sequestosome-1 (SQSTM1), a ubiquitin-binding protein p62, is a protein of the autophagosome cargo that tags other proteins for differentiated autophagy. During autophagy, SQSTM1 is degraded. Both LC3II/I and SQSTM1 ratios are widely used as indicators of autophagy [148,149,150]. Zebrafish and sea bass were exposed to PS-MPs, and the LC3 II/I ratio was increased, and SQSTM1/p62 levels were decreased in the livers of both fish after exposure to PS-MPs, compared with the controls [131]. This result suggests the development of hepatocyte autophagy.

Embryonic zebrafish fibroblast cell lines (ZF4) were exposed to PS-NPs at 100 and 1000 nm, and confocal images showed that the NPs of both sizes were deposited in the lysosomes but could escape through the lysosomal rupture. The subsequent deposition of 100-NPs in the cytoplasm leads to the loss of mitochondrial membrane potential and the massive production of reactive oxygen species, which ultimately stimulates the activation of caspases, disrupts mitophagy, and leads to irreversible cell death [151].

In contrast, the toxicity of 1000-NPs to ZF4 cells did not involve the loss of lysosomal permeability and mitochondrial membrane potential. This large-sized nanoplastic lysosomal deposition mainly induces lysosomal acidification, activates autophagy, and disrupts the integrity of cell membranes.

Immunohistochemical results [152] showed that the expression of autophagy-associated tubulin (Tub), microtubule-associated protein light chain 3 (LC3), and p62 (Sequestosome 1) increased after the exposure of the marine polychaete Hediste diversicolor to different environmental MPs collected from the southern Mediterranean coast, suggesting that MPs activates the autophagy system of marine hairy organisms.

PS-MPs were also found to activate the expression of autophagy-related proteins in chicken cerebellum and avian heart, respectively, in chickens [153] and birds [154].

#### 5.6.3. Endoplasmic Reticulum Stress

Endoplasmic reticulum stress can trigger and regulate autophagy [155]. In the PERK pathway, autophagy can be induced through the PERK/eIF2α/ATF4 pathway, or PERK can directly activate autophagy-related gene expression to mediate autophagy [156]. ER stress occurs when GRP78 dissociates from the aforementioned transmembrane proteins and binds with high affinity to accumulated mis/unfolded proteins, while IRE1 and PERK dissociated from GRP78 are activated by trans-autophosphorylation. The dissociated ATF6 is activated by proteolysis, thereby inducing the expression of downstream signaling pathways and the related genes LC3, P62, ATGs, and Beclin1, and finally activating the autophagy pathway [157].

The exposure of mice to MPs induces endoplasmic reticulum stress in the liver. The mRNA levels of the endoplasmic reticulum stress pathway-related markers PERK and CHOP were both increased after MPs exposure, while MPs exposure significantly increased the protein expression levels of p-PERK, p-eIF2α, ATF4, and CHOP in the liver, indicating that MPs can activate the eIF2α-ATF4-CHOP axis in the liver to induce endoplasmic reticulum stress [133].

**Figure 6:**
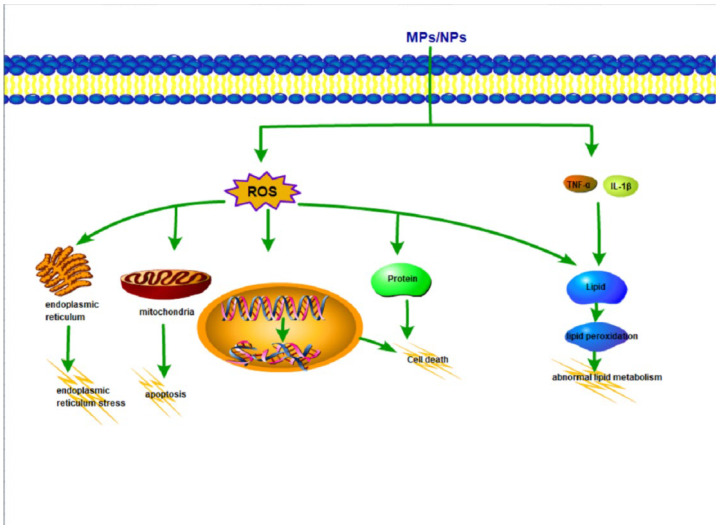
The interrelated cellular parameters.

To sum up, the cell mechanism diagram is as follows (Figure 6).

## 6. Conclusions

### 6.1. Animal Health

With the increasing application of plastic products and human exposure, people have gradually begun to pay attention to the adverse effects caused by plastic products. The liver is the body’s largest organ responsible for detoxification and metabolism and undertakes many important activities. The toxic effects of MPs on the liver are receiving more attention from researchers. Currently, the research on the toxic effects of MPs/NPs on the liver mainly focuses on marine fish [69,124]. Since the pollution of MPs in the ocean is not optimistic, and fish are the main marine species that people eat, it is crucial to study the impact of MPs on marine fish. However, MPs are not only present in the ocean [158] but also can be detected in soil [159] and air [160]; thus, it is necessary to study the damage of MPs to mammalian livers. Investigations have been conducted on the effects of MPs on marine fish and mammals; however, more studies are needed to provide scientific theoretical support for plastic control.

### 6.2. Human Health

People can ingest MPs/NPs from the external environment through diet and breathing. The main way of diet is to consume seafood [161], mainly shellfish such as fish [162,163] and oysters [164]. In addition, MPs/NPs were also detected in many foods, such as sugar, honey [18], salt [165], etc. These foods are closely related to human life and deserve our attention.

Studies have shown that MPs/NPs also exist in the air. Although the exposure concentration of MPs/NPs in the air is relatively low, long-term exposure at low concentrations may also cause potential harm to human health. Some occupational groups (such as the synthetic textile industry and plastic industry) are exposed to high concentrations of MPs/NPs every day and are more vulnerable to MPs/NPs than the normal population [16].

### 6.3. What Can We Do in the Future?

The current research on the effects of MPs on liver toxicity still has the following limitations:

Toxicity of MPs: In the real environment and process of natural degradation, plastic is subjected to its interactions with physical, chemical, and biological factors [166,167]; thus, the properties of MPs have changed, and the surface can adsorb various persistent organic pollutants [168], heavy metals [169], etc., which will modify the toxicity of MPs, meaning that MPs in the environment are different from the single microplastic prepared by the company that is used in most experiments [170]. At present, there are some studies on MPs combined with other toxicants [171,172], and some MPs are derived from naturally degraded plastics [173] in the environment. Although the experiment is complicated, it is of practical significance.

Exposure dose of MPs: The toxicity of MPs depends on many aspects, including the particle size [138], concentration [42], and exposure duration [128] of MPs. Compared with the plastic concentrations in the environment, the doses of MPs used in many studies are excessively large. The highest abundance of MPs/NPs in the ocean can reach 8.6 × 10^3^ particles/m^3^, and surveys have found that people ingest 39,000–52,000 particles per year on average [24]. Based on this, it is possible to estimate the difference between the doses of MPs/NPs used in the study and the MPs/NPs content in the ocean, and the average annual intake of MPs/NPs by humans. Oryzias melastigmas were exposed to MPs at 1.82 × 10^10^ particles/m^3^ [48], which is 7 orders of magnitude higher than MPs/NPs in the ocean. Similar results were found in the experiments of Lu, Y. [27] and Ding, J. et al. [44]. In mammals such as mice, the daily dose used in one study [64] contained 2.27 × 10^4^ MPs, which is almost half of the annual human exposure (that is, if the daily dose of mice is given to humans for one year, the annual total particle intake would be at least two orders of magnitude higher than the estimated actual human annual intake). Additionally, the smaller the particle size of MPs, the higher the number of particles contained. In the same study [64], there were 1.46 × 10^6^ particles in the daily exposure dose of 5 μm MPs. According to the same method, the daily exposure dose w two orders of magnitude higher than the human exposure dose in one year! (i.e., if daily doses in mice were given to humans for a year, the total annual particle intake would be at least orders of magnitude higher than the estimated actual annual intake in humans).

Considering that the body is exposed to MPs/NPs in a variety of ways and can accumulate at a high trophic level [174,175,176], many studies have not given the number of particles, which cannot be compared with the content of MPs/NPs in the environment [28,45]. Moreover, in the investigation and research on the abundance of MPs/NPs in water, the investigation methods are not uniform, resulting in inconsistent research units. For example, when trawls are used to sample large-scale water bodies, the MPs/NPs unit are usually expressed as “pieces/km^2^”, while collecting water samples with buckets, the unit of MPs/NPs are expressed as “pieces/m^3^” [177].

Similarly, the abundance of MPs/NPs in sediments includes different representations per unit weight (units/kg) and unit area (units/m^2^) [178], while weight also includes dry and wet weights. In addition, the inconsistency of research methods also affects the reliability of the obtained data and the horizontal comparison of these data; this problem is more prominent in the research on air MPs/NPs that started later [12,179]. Therefore, there is an urgent need to establish a unified standard to quantify microplastics and to compare the doses used in experimental studies with realistic MPs levels.

NPs and MPs: To date, many studies focus on the toxic effects of MPs on the liver, and NPs have unique characteristics, which have stronger effects in inducing the production of ROS in the liver and development of oxidative stress and inflammation [87], and more research should be conducted on the effects of NPs.

Types of MPs: The current research on MPs mainly focuses on polystyrene MPs [87,180]. Polystyrene plastics are widely used in people’s daily life, but the results of several surveys [181,182,183] show that polyethylene, polyamide, and polyethylene terephthalate MPs are the most abundant in the stomach and liver of marine fish. The shape is mostly fibrous. Subsequent research should be based on real environmental situations, and some other types and shapes of MPs should be studied.

Hepatotoxicity of MPs: The research on the toxicity of MPs to the liver mainly focuses on inflammation and oxidative stress, and the possible mechanisms of MPs on liver damage should be further explored to provide scientific theory and foundation for the prevention and control of MPs.

This article systematically summarizes the accumulation of MPs/NPs in the liver, and the effects on liver pathology and liver function, and discusses the possible underlying mechanisms to provide clues to the liver injury caused by MPs or NPs. It also provides a scientific basis for future research directions.

## Figures and Tables

**Table 1:** The study parameters and effects of accumulation of NPs in the liver.

Research Object	Particle Size	Material	Number of Particles	Concentration	Mode of Exposure	Exposure Time	Summary	Reference
Wistar male rats	25, 50 nm	PS	-	1, 3, 6, 10 mg/kg bw/day	Oral gavage	5 weeks	In the high-dose gavage group, the accumulation of PS NPs in the liver was confirmed by whole-body scanning.	[28]
Zebrafish	42 nm	PS	-	1 mg/g bw	In diet	7 days	The co-parentally exposed F1 larvae at 120 hpf had a significant amount of fluorescence in the liver, indicating that PS-NPs accumulate in the liver of zebrafish larvae.	[45]
Zebrafish liver cells	65 nm	PS	-	5, 50 mg/L	In culture medium	6–72 h	100% of ZFL cells took up the fluorescent PS-NPs after 6 h of incubation. Additionally, the ZFL internalization dynamics followed a dose–response pattern, with cells incubated at higher doses and longer times presenting higher fluorescence.	[42]
Zebrafish	70 nm	PS	-	0.5, 1.5, 5 ppm	In water	30 days	After 30 days of incubation with 1.5 ppm florescence PS-NPs, green fluorescence was seen in the liver.	[51]
Hepatocytes of the large yellow croaker	80 nm	PS	-	1, 10, 100 mg/L	In diet	3, 12 h	Results showed that PS-NPs could be accumulated in hepatocytes. Moreover, the uptake of PS-NPs by hepatocytes increased with the increase in the time and concentrations of PS-NPs treatment.	[52]
HL7702 cells	100 nm	PS	-	1 mg/L	In culture medium	24 h	There was a large number of PS-NPs in the cytoplasm.	[46]
C57 male mice	100 nm	PS	-	0.1, 1 mg/L	In water	60 days	High-dose group of PS-NPs accumulated in mouse liver.	[46]
Goldfish	250 nm	PS	-	300 mg/L	In water	168 h	PS-NPs could accumulate in the liver, increasing over time within 7 days.	[47]
Mice	200~300 nm	PS	-	578 μg/mL	Oral gavage	48 h	Uptake of [^64^Cu] Cu-DOTA-polystyrene was observed in the liver at 48 h after administration.	[53]
CD-1 female mice	790 nm	PS	-	30 mg/kg	Oral gavage	35 days	The concentration of PS-NPs in the liver was (69.86 ± 25.31) μg/g.	[54]

hpf: hours post-fertilization; bw: body weight; -: unknown.

**Table 2:** The study parameters and effects of accumulation of MPs on the liver.

Research Object	Particle Size	Material	Number of Particles	Concentration	Mode of Exposure	Exposure Time	Summary	Reference
Zebrafish	2.34 ± 0.07 μm	PLABio MPs	-	2.5, 5 mg/L	In water	30 days	PLABio MPs at concentrations of 2.5 and 5 mg/L could accumulate in the liver.	[45,55]
Zebrafish	5 μm	PS	2.9 × 10^2^, 2.9 × 10^3^, 2.9 × 10^4^ particles/mL	20, 200, 2000 μg/L	In water	7 days	PS-MPs accumulated in the fish liver after 7 days of exposure.	[27]
Male crab C. japonica	5 μm	PS	1.0 × 10^3^ particles/mL	0.68 × 10^−4^ mg/mL	In water	7 days	PS-MPs accumulated the most in crab hepatopancreas.	[56]
Male mice	5, 20 μm	PS	1.46 × 10^6^ items (5 μm), 2.27× 10^4^ items (20 μm)	0.1 mg/day	Oral gavage	28 days	After 4 weeks of exposure, the maximum tissue concentration of PS-MPs in the liver was 0.303 ± 0.029 mg/g.	[29]
Marine medaka	10 μm	PS	-	2, 20, 200 μg/L	In water	60 days	PS-MPs were accumulated in the liver of marine medaka at all exposure doses.	[53,57]
Marine medaka	10 μm	PS	1.82 × 10^10^ particles/m^3^	10 mg/L	In water	30–60 days	It was observed that over 30 spherical PS with a diameter ≤3 μm accumulated in the exposed group.	[48]
Male Swiss mice	35.46 ± 18.17 μm	PE	-	60 mg/L	Oral gavage	7 days	PE-MPs could accumulate in the mouse liver.	[54,58]
Red tilapia	70~90 μm	PS	3.51 × 10^4^ particles/mL	100 μg/L	In water	14 days	PS-MPs could accumulate in the tilapia liver, and it showed a generally increasing tendency with time.	[44]

PLABio MPs: polylactic acid bio microplastics; -: unknown.

**Table 3:** The study parameters and effects of hepatic pathomorphological changes caused by NPs on the liver.

Research Object	Particle Size	Material	Number of Particles	Concentration	Mode of Exposure	Exposure Time	Summary	Reference
C57BL/6 female mice	42 nm	PS	-	10, 50 μg/mL	Inject via tail vein	5 injections in 15 days	Hepatocyte binucleation increased after exposure. Fatty degeneration and ballooning were significantly increased in the liver tissue of the high-fat-fed mice, and the perilobular steatosis was severe.	[68]
Fish	60 nm	PS	-	5 mg/L	In water	7 days	Hepatocytes in the exposed group were destroyed with aggregated and condensed nuclei.	[5]
Zebrafish	70 nm	PS	1.1 × 10^8^, 1.1 × 10^9^, 1.1 × 10^10^ particles/mL	20, 200, 2000 μg/L	In water	3 weeks	Necrosis, infiltration, and fat droplets were observed in hepatocytes.	[27]
Male C57 mice	100 nm	PS	-	0.1, 1 mg/L	In water	60 days	Hepatocellular edema and vacuolar degeneration, enlarged nucleus, cell dikaryon, irregularly arranged hepatic cords, the proliferation of bile ducts, as well as the inflammation of portal areas were found in the PS-NPs exposed groups.	[46]
Juvenile groupers	100.86 ± 7.15 nm	PS	-	300, 3000 μg/L	In water	14 days	Hepatocyte vacuolization was observed in the 300 and 3000 μg/L exposure groups.	[69]
Zebrafish	100~120 nm	PS	-	10, 100 μg/L	In water	35 days	PS-MPs caused histopathological damage such as inflammation, degeneration, and hemorrhage in zebrafish liver tissue.	[66]
Larimichthys polyactis	190 nm	PS	-	1 mg/L	In diet	8 days	There was some zonal necrosis, a decrease in tissue density, and normal-stained nuclei in the liver of the NPs-exposed fish.	[70]
Goldfish	250 nm	PS	-	0, 0.05, 0.5, 5 mg/L	In water	28 days	Necrosis, cellular swelling, and hemorrhage were observed in the PS-NPs-exposed liver.	[47]

-: Unknown.

**Table 4:** The study parameters and effects of hepatic pathomorphological changes caused by MPs on the liver.

Research Object	Particle Size	Material	Number of Particles	Concentration	Mode of Exposure	Exposure Time	Summary	Reference
Zebrafish	5 μm	PS	2.9 × 10^2^, 2.9 × 10^3^, 2.9 × 10^4^ particles/mL	20, 200, 2000 μg/L	In water	3 weeks	Necrosis, infiltration, and fat droplets were observed in PS-MPs exposed hepatocytes.	[27]
Goldfish	5 μm	PS	-	10, 100, 1000 μg/L	In water	7 days	After exposure in the high-dose group, there was blood cell infiltration in the liver of the goldfish.	[63]
ICR mice	5 μm	PS	-	100, 1000 μg/L	In water	6 weeks	Liver pathological sections showed increased liver ballooning degeneration in mice.	[65]
C57 male mice	5 μm	PS	1.46 × 10^6^ particles	20 mg/kg bw/day	In water	30 days	The PS-MPs exposed group showed severe vacuolar degeneration, chronic inflammatory infiltration, and hepatocyte edema.	[64]
Goldfish	8 μm	PS	-	0, 0.05, 0.5, 5 mg/L	In water	28 days	Necrosis, cellular swelling, and hemorrhage were observed in PS-MPs-exposed liver.	[47]
Marine medaka	10 μm	PS	-	2, 20, 200 μg/L	In water	60 days	Compared with the control group, a significant decrease in the hepatosomatic index was found in adult male marine medaka exposed to PS-MPs.	[57]
Marine jacopever	15 μm	PS	1 × 10^6^ microspheres/L	-	In water	7 days of decontamination after 14 days of exposure	The Liver pathological section showed hyperemia in the PS-MPs exposed group.	[59]
Grouper	22.3 μm	PS	-	2, 20 mg/g df	In water	25 days	Eosinophilic infiltration was observed in the exposed liver.	[50]
Tadpoles	35.46 ± 18.17 μm	PE	4.24 × 10^−6^ particles/m^3^	60 mg/L	In water	7 days	Vasodilation, infiltration, hyperemia, edema degeneration, hypertrophy, and hyperplasia occurred in the exposed liver.	[61]
Clarias gariepinus	95.41 ± 4.23 μm	PVC	-	0.5, 1.5, 3.0%	In diet	30 days of decontamination after 45 days of exposure	Hepatocyte necrosis, fat vacuolization and degeneration, and glycogen depletion were observed in PVC group.	[62]
European sea bass	40~150 μm	PVC/PE	-	0, 100, 500 mg/kg·di	In diet	3 weeks	Hepatocytes showed vacuolation, infiltration, and focal necrosis after exposure to MPs.	[60]

-: Unknown; bw: body weight; df: dry food; di: diet.

**Table 5:** The study parameters and effects of MPs and NPs on liver inflammation.

Research Object	Particle Size	Material	Number of Particles	Concentration	Mode of Exposure	Exposure Time	Summary	Reference
Zebrafish	70 nm, 5 μm	PS	1.1 × 10^8–10^ particles/mL (70 nm), 2.9 × 10^2–4^ particles/mL (5 μm)	0, 20, 200, 2000 μg/L	In water	3 weeks	Histopathological analysis showed that inflammation occurred in the liver of zebrafish in the 70 nm and 5 μm 2000 μg/L PS-MP groups.	[27]
Nile tilapia	350 μm, 9 μm	PS	-	5 mg/L	In water	28 days	Expression of interferon-γ (IFN-γ) genes was upregulated in the livers of fish exposed to 0.35 μm and 9 μm compared with controls; interleukin 8 (IL8), interleukin (IL-1β), and tumor necrosis factor (TNF-α) gene expression was upregulated in the 9 μm group.	[97]
Gilthead seabream	100~500 μm	PE	-	10%	In diet	90 days (30 days of purification)	The level of inflammatory factor MPO in the liver of the exposed group increased.	[100]
Sparus aurata Linnaeus	200~500 μm	PE	-	10%	In diet	90 days (30 days of purification)	Exposure induced an inflammatory response in the liver, manifested by elevated MPO levels.	[75]
Zebrafish	100~120 nm	PS	-	0, 10, 100 μg/L	In water	35 days	PS-MPs caused inflammatory damage in zebrafish liver tissue.	[66]
Javanese medaka fish	5 µm	PS	1.46 × 10^3~5^ particles	0, 100, 500, 1000 µg/L	In water	21 days	Significant inflammatory changes were observed in the liver.	[101]
Oryzias melastigma	2, 10, 200 μm	PS	-	10 mg/L	In water	60 days	Fish in the 2 and 10 μm MPs-exposed groups exhibited liver damage, mainly manifested by the presence of inflammation.	[102]
Male mice (Mus musculus, ICR)	5, 20 μm	PS	1.46 × 10^6^ items (5 µm) 2.27 × 10^4^ items (20 µm)	0.01, 0.1, 0.5 mg/day	Oral gavage	28 days	Two particle sizes of liver HE staining showed inflammation.	[29]
ICR mice	-	PS	-	0, 5, 25, 50 mg/kg bw	Oral gavage	2 weeks	In the liver of NP-treated mice, the expression of inflammatory response proteins (iNOS, COX-2) and the mRNA levels of inflammatory cytokines were significantly increased.	[103]
High-fat diet C57BL/6 female mice	42 nm	PS	-	0, 10, 50 μg/mL	Inject via tail vein	5 times in 15 days	Liver Kupffer cell (KC) infiltration was enhanced, and proinflammatory factor expression was elevated.	[68]
Mice	5 μm	PS	1.46 × 10^6^ particles	20 mg/kg bw/day	In water	30 days	The liver in the mic-P group showed severe vacuolar degeneration, chronic inflammatory infiltration, and hepatocyte edema.	[64]
ICR male mice	5 μm	PS	-	0, 0.1, 0.5, 1 mg/mL	In water	4 weeks	The expression of interleukins IL-1β and IL-18 increased in the microplastic exposure group.	[104]
C57BL/6J mice	500 nm	PS	-	0.5 mg/100 µL	In water	4 weeks	MPs upregulated the mRNA expressions of inflammatory factors *IFN-γ*, *TNF-α*, *IL-1β*, *IL-6,* and *IL-33*, and downregulated *IL-4*, *IL-5*, *IL-10*, *IL-18*, and *TGF-β1* in the liver.	[98]
C57 mice	5 μm	PS	-	500 μg/L	In water	28 days	Exposure to MP results in the expression of inflammatory factors in the liver.	[105]

-: Unknown; bw: body weight.

**Table 6:** The study parameters and effects of MPs and NPs on hepatic lipid metabolism.

Research Object	Particle Size	Material	Number of Particles	Concentration	Mode of Exposure	Exposure Time	Summary	Reference
Zebrafish	70 nm, 5 μm	PS	1.1 × 10^8–10^ particles/mL, 2.9 × 10^2–4^ particles/mL	0, 20, 200, 2000 μg/L	In water	3 weeks	Lipid droplets were found in the liver pathological pictures of the 2000 μg/L 70 nm, 5 μm exposure group.	[27]
Juvenile D. labrax	45 nm	PMMA	-	0, 0.02, 0.2, 2, 20 mg/L	In water	96 h	The transcription levels of the genes related to lipid metabolism, *pparα*, *pparγ* (*peroxisome proliferator*-*activated receptor*), and *nd5*, were upregulated in the liver at 96 h exposure.	[107]
Black rockfish	500 nm, 15 μm	PS	-	190 μg/L	In water	21 days	The HIS (liver index) value was generally positively correlated with the liver metabolism (e.g., lipid, protein) of the fish, and the 15μm group had a larger liver index than the other groups.	[106]
Larimichthys crocea	80 nm	PS	-	0, 1, 10, 100 mg/kg·di	In diet	21 days	The liver of the high-dose group was enlarged and appeared white, and the contents of TG and lipids in the liver after PSNPs exposure were significantly higher than those in the control group. Oil red O staining showed that the higher the exposure dose, the higher the accumulation of lipid droplets in the liver; the expression of the genes involved in the process of lipid synthesis, decomposition, and transport changed.	[52]
Yellow croaker hepatocytes	80 nm	PS	-	0, 5, 20, 80 mg/L	In culture medium	24 h	The expression of lipid synthesis genes *fas*, *srebp1,* and *pparγ* in hepatocytes increased, and the lipid catabolism *atg1*, *pparα,* and *aco* genes increased and then decreased.	[52]
Oryzias melastigmas	10, 200 μm	PS	1.82 × 10^10^ particles/m^3^, 2.27 × 10^6^ particles/m^3^	10 mg/L	In water	30, 45, 60 days	PS exposure inhibited the accumulation of fatty acid, fatty acid methyl ester, and fatty acid ethyl ester in the liver of marine medaka.	[48]
Oryzias melastigma	2, 10, 200 μm	PS	-	10 mg/L	In water	60 days	The hepatic lipid content was significantly increased in the 200 μm PS-MPs exposure group.	[102]
ICR male mice	5, 20 μm	PS	1.46 × 10^6^ items, 2.27 × 10^4^ items	0.01, 0.1, 0.5 mg/day	Oral gavage	28 days	Hepatic HE staining showed lipid droplets; the levels of TC and TG in the liver decreased.	[29]
ICR mice	5 μm	PS	-	0, 100, 1000 μg/L	In water	6 weeks	The levels of TCH and TG in the liver of the parental female mice were significantly higher in a concentration-dependent manner than those of the control group, and the level of TCH in the F1 liver was significantly decreased; the level of TG in the liver of the F1 male mice changed, and the level of TG in the F1 female mice decreased;	[65]
Mice *Mus musculus*	5, 20 μm	PS	-	0, 0.01, 0.1, 0.5 mg/mL	Oral gavage	4 weeks	Exposure to 5 and 20 μm PS-MPs inhibited liver TG levels in mice.	[115]
High-fat diet C57BL/6 female mice	42 nm	PS	-	0, 10, 50 μg/ml	Inject via tail vein	5 times in 15 days	Liver TC, TG, *pparα*, and *pparγ* gene mRNA expression were not affected, unlike the *fat* and *cpt1α* gene levels, which were.	[68]
C57 mice	5 μm	PS	-	500 μg/L	In water	28 days	Exposure to PSMP caused TG accumulation.	[105]

PMMA: polymethyl–methacrylate; di: diet; -: unknown.

**Table 7:** The study parameters and effects of MPs and NPs on hepatic energy metabolism.

Research Object	Particle Size	Material	Number of Particles	Concentration	Mode of Exposure	Exposure Time	Summary	Reference
Zebrafish	70 nm, 5,	PS	1.1 × 10^8–10^ particles/mL, 2.9 × 10^2–4^ particles/mL	0, 20, 200, 2000 μg/L	In water	3 weeks	Energy metabolism is altered in zebrafish liver after exposure to MPs.	[27]
ICR male mice	5, 20 μm	PS	1.46 × 10^6^ items, 2.27 × 10^4^ items	0.01, 0.1, 0.5 mg/day	Oral gavage	28 days	After exposure to MPs, ATP decreased and LDH levels increased in the livers of mice.	[29]
ICR mice	5 μm	PS	-	0, 100, 1000 μg/L	In water	6 weeks	MPs affect many activities in mouse livers related to energy metabolism.	[65]
Black rockfish	500 nm, 15 μm	PS	-	190 μg/L	In water	21 days	MPs affect crude protein and crude fat content in fish livers.	[106]
Zebrafish	70 nm	PS	-	0.5, 1.5, 5 ppm	In water	30 days	NPs affect energy metabolism in zebrafish liver.	[51]
Grass carp	32~40 μm	PS	-	100, 1000 μg/L	In water	21 days	The results of pathway analysis showed that MPs affected the signaling pathways related to energy metabolism in grass carp liver.	[127]
Minnows	1 μm	PS	(3.71 ± 0.1) × 10^8^ items/L	200 μg/L	In water	28 days	MP exposure interferes with hepatic energy metabolism in the minnow.	[124]

-: Unknown.

**Table 8:** The study parameters of MPs and NPs in liver apoptosis.

Research Object	Particle Size	Material	Number of Particles	Concentration	Mode of Exposure	Exposure Time	Reference
Grouper	22.3 μm	PS	-	2, 20 mg/g·dt	In water	25 days	[50]
Zebrafish, perch	5~12 μm	PS	-	0.0075/0.85 g/day	In water	21 days	[131]
Mice	5 μm	PS	1.46 × 10^6^ particles	20 mg/kg·bw/day	In water	30 days	[64]
Goldfish	1 μm	PS	0, 10, 100, 1000 particles/mL	-	In water	7 days	[128]
Male mice	5 μm	PS	1.46 × 10^6^ particles	0.1 mg/day	In water	90 days	[133]
SMMC-7721	500 nm	PS	-	20 μg/mL	In culture medium	24 h	[129]
Zebrafish	50,100 nm	PS	-	0.1, 0.5, 2, 10 mg/L	In water	120 hpf	[137]
Zebrafish, perch	10~45 μm 106~125 μm	PE	-	10 mg/g·df	In water	21 days	[138]
RAW264.7	42 nm	PS	-	0, 1, 5, 10 mg/mL	In water	24 h	[139]

Dt: dry tissue; bw: body weight; -: unknown; df: dry food.

## Supplementary Materials

The following supporting information can be downloaded at: https://www.mdpi.com/article/10.3390/toxics10100586/s1, Table S1: word frequency table for keyword analysis.
